# BAG3 promotes autophagy and glutaminolysis via stabilizing glutaminase

**DOI:** 10.1038/s41419-019-1504-6

**Published:** 2019-03-25

**Authors:** Song Zhao, Jia-Mei Wang, Jing Yan, Da-Lin Zhang, Bao-Qin Liu, Jing-Yi Jiang, Chao Li, Si Li, Xiao-Na Meng, Hua-Qin Wang

**Affiliations:** 1 Department of Biochemistry & Molecular Biology, China Medical University, Shenyang, 110026 China; 2 Key Laboratory of Cell Biology, Ministry of Public Health, and Key Laboratory of Medical Cell Biology, Ministry of Education, China Medical University, Shenyang, 110026 China; 3 Institute of Life Sciences, Jinzhou Medical University, Jinzhou, 121001 China; 4 Department of Thyroid Surgery, The 1st Affiliated Hospital, China Medical University, Shenyang, 110001 China

## Abstract

Bcl-2 associated athanogene 3 (BAG3) is an important molecule that maintains oncogenic features of cancer cells via diverse mechanisms. One of the important functions assigned to BAG3 is implicated in selective macroautophagy/autophagy, which attracts much attention recently. However, the mechanism underlying regulation of autophagy by BAG3 has not been well defined. Here, we describe that BAG3 enhances autophagy via promotion of glutamine consumption and glutaminolysis. Glutaminolysis initiates with deamination of glutamine by glutaminase (GLS), by which yields glutamate and ammonia in mitochondria. The current study demonstrates that BAG3 stabilizes GLS via prohibition its interaction with SIRT5, thereby hindering its desuccinylation at Lys158 and Lys164 sites. As an underlying molecular mechanism, we demonstrate that BAG3 interacts with GLS and decreases SIRT5 expression. The current study also demonstrates that occupation by succinyl at Lys158 and Lys164 sites prohibits its Lys48-linked ubiquitination, thereby preventing its subsequent proteasomal degradation. Collectively, the current study demonstrates that BAG3 enhances autophagy via stabilizing GLS and promoting glutaminolysis. For the first time, this study reports that succinylation competes with ubiquitination to regulate proteasomal GLS degradation.

## Introduction

Bcl-2-associated athanogene 3 (BAG3) is a member of protein heat-shock protein (HSP70) co-chaperones that interact with the ATPase domain of HSP70 through a conserved C-terminal BAG domain^1^. To data, six human BAG members (BAG1-6) have been identified, and BAG3 attracts much attention because of its modular structure: a WW domain at the N-terminus, two IPV domains which can interact with HspB6 and HspB8, a proline-rich region (PxxP) in the center of the protein, and a conserved BAG domain at the C-terminus^2^. BAG3 executes multiple physiological and pathological functions, and one of the important functions assigned to BAG3 is related to its involvement in protein homeostasis by regulation of selective macroautophagy/autophagy under stressful conditions^3–14^. Autophagy is an evolutionarily conserved catabolic process that is important to maintain cellular homeostasis^15^. Although autophagy was considered to be a random process for many years, accumulating data now support that it is a selective process and receives tight regulation^16^. It has been well documented that BAG3 is induced by various stressful stimuli and facilitates selective autophagy to serve as an adaptive response to maintain cellular homeostasis^7,8,10,13,17–22^. However, the molecular mechanism(s) underlying regulation of autophagy by BAG3 are not yet fully elucidated.

Glutamine is the most abundant amino acid in the plasma and converted to glutamate and further to alpha-ketoglutarate (α-KG) to enable ATP production through the tricarboxylic acid (TCA) cycle^23,24^. Glutaminolysis is a metabolic pathway that starts with deamination of glutamine by glutaminase (GLS) to yield glutamate and ammonia in mitochondria^25^. There are two forms of GLS in humans: kidney-type glutaminase (GLS, KGA or GAC) and liver-type glutaminase (GLS2, LGA or GAB). GLS is expressed ubiquitously, whereas GLS2 is expressed primarily in the liver^26^. GLS is often overexpressed in a wide variety of tumors and its upregulation has been reported to correlate with tumor growth^27^. Glutaminolysis takes place in all proliferating cells and plays a critical role in maintaining bioenergetics and providing nitrogen, sulfur and carbon skeletons for macromolecular biosynthesis^23,24^. Glutaminolysis also plays an important role in regulating redox balance, mTOR signaling^28–31^. In addition, glutaminolysis is an important source of cellular ammonia^32,33^, which induces autophagy in tumor cells independent of mTOR and ULK1/2^31,33,34^.

The current study demonstrates that BAG3 promotes autophagic activity via enhancing glutaminolysis and ammonia generation. In terms of mechanism, our results show that BAG3 enhances succinylation of GLS at Lys158 and Lys164 sites, which suppressed its Lys48-linked ubiquitination and subsequent proteasomal degradation.

## Results

### Ectopic BAG3 expression induces autophagy

**Fig. 1:**
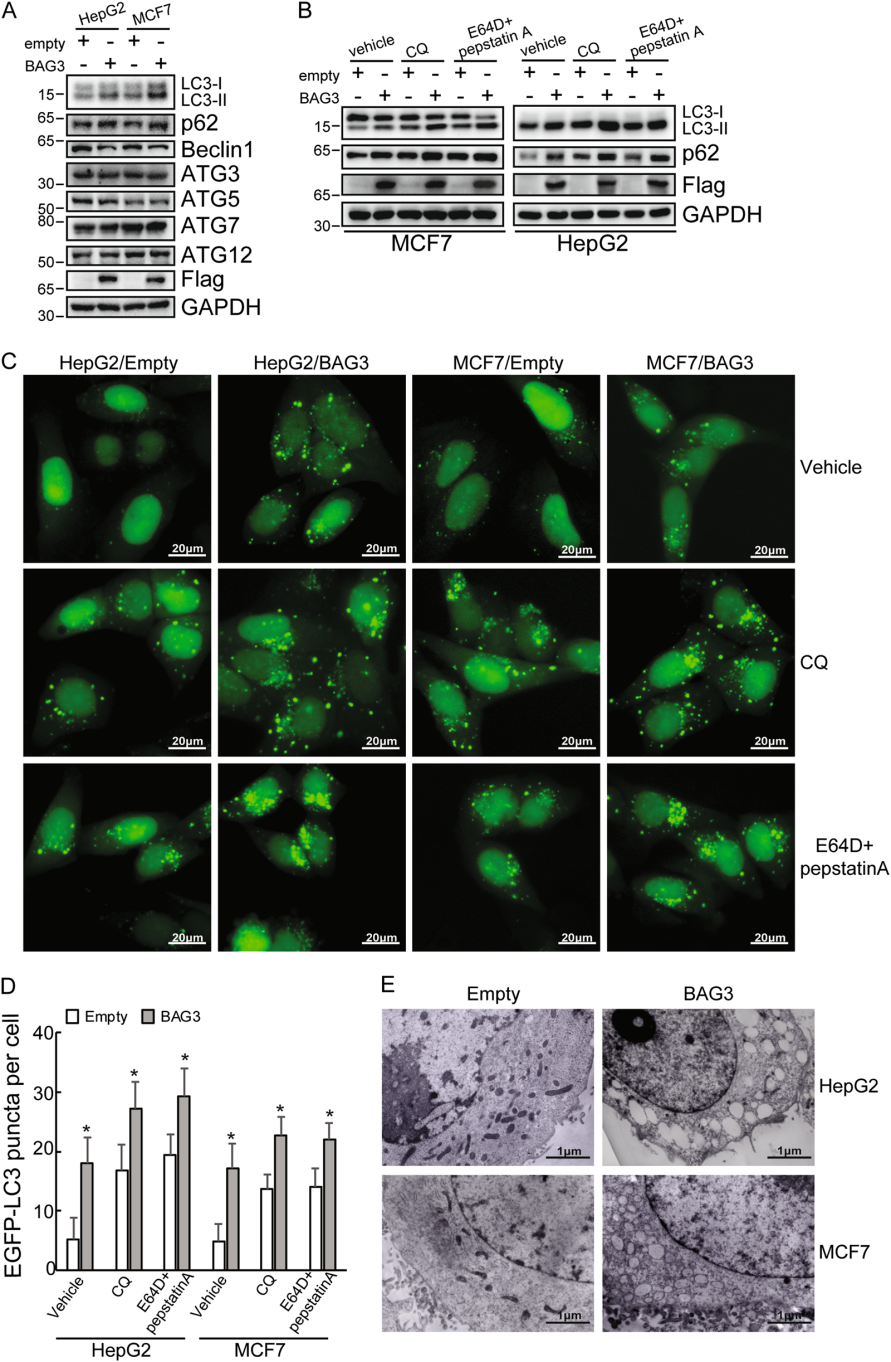
Ectopic BAG3 expression induces autophagy. **a** HepG2 or MCF7 cells were infected with lentivirus containing empty or BAG3 construct. Total protein was extracted and protein expression of indicated autophagy-related genes were investigated by immunoblotting. **b** HepG2 or MCF7 cells infected with lentivirus containing empty or BAG3 construct were treated with vehicle, CQ and E64D plus pepstatin A respectively, protein expression levels of LC3, p62 and BAG3 were analyzed using Western blot analysis. **c** HepG2 or MCF7 cells stably overexpressing EGFP-LC3B were infected with lentivirus containing empty or BAG3 construct. Cells were treated with vehicle, CQ or E64D plus pepstatin A, and the punctate distribution of EGFP-LC3B was visualized under the florescence microscopy. **d** Quantitative analysis of (**c**). Results shown represent mean ± SD from five representative microscopic fields and three independent experiments were performed. **e** HepG2 or MCF7 cells transduced with lentivirus containing empty or BAG3 construct, and ultrastructure was analyzed using transmission electron microscopy. *P < 0.05. Error bars indicate means ± SD

BAG3 was ectopically expressed in two cell lines HepG2 and MCF7, which were frequently utilized as tools for autophagy study. Western blot demonstrated that ectopic BAG3 expression increased LC3-II and p62, while decreased Beclin 1 expression (Fig. 1a). The protein expression levels of ATG3, ATG5, ATG7 and ATG12 were unaltered by ectopic BAG3 expression (Fig. 1a). Blocking autophagy at late stage using chloroquine (CQ) or E64D and pepstatin A markedly increased LC3-II and p62 levels, indicating that BAG3 indeed increased autophagic flux in HepG2 and MCF7 cells (Fig. 1b). EGFP-LC3B stable expression cells were also generated. BAG3 significantly increased puncta distribution of EGFP-LC3B, which was further increased by CQ or E64D and pepstatin A (Fig. 1c, d). Ultrastructural observation using transmission electron microscopy observed obvious accumulation of small vacuoles in the cytoplasm of cells with ectopic BAG3 expression (Fig. 1e). These data indicated that BAG3 increased autophagy.

### BAG3 overexpression induces autophagy independent of Beclin 1 and PtdIns3K

**Fig. 2:**
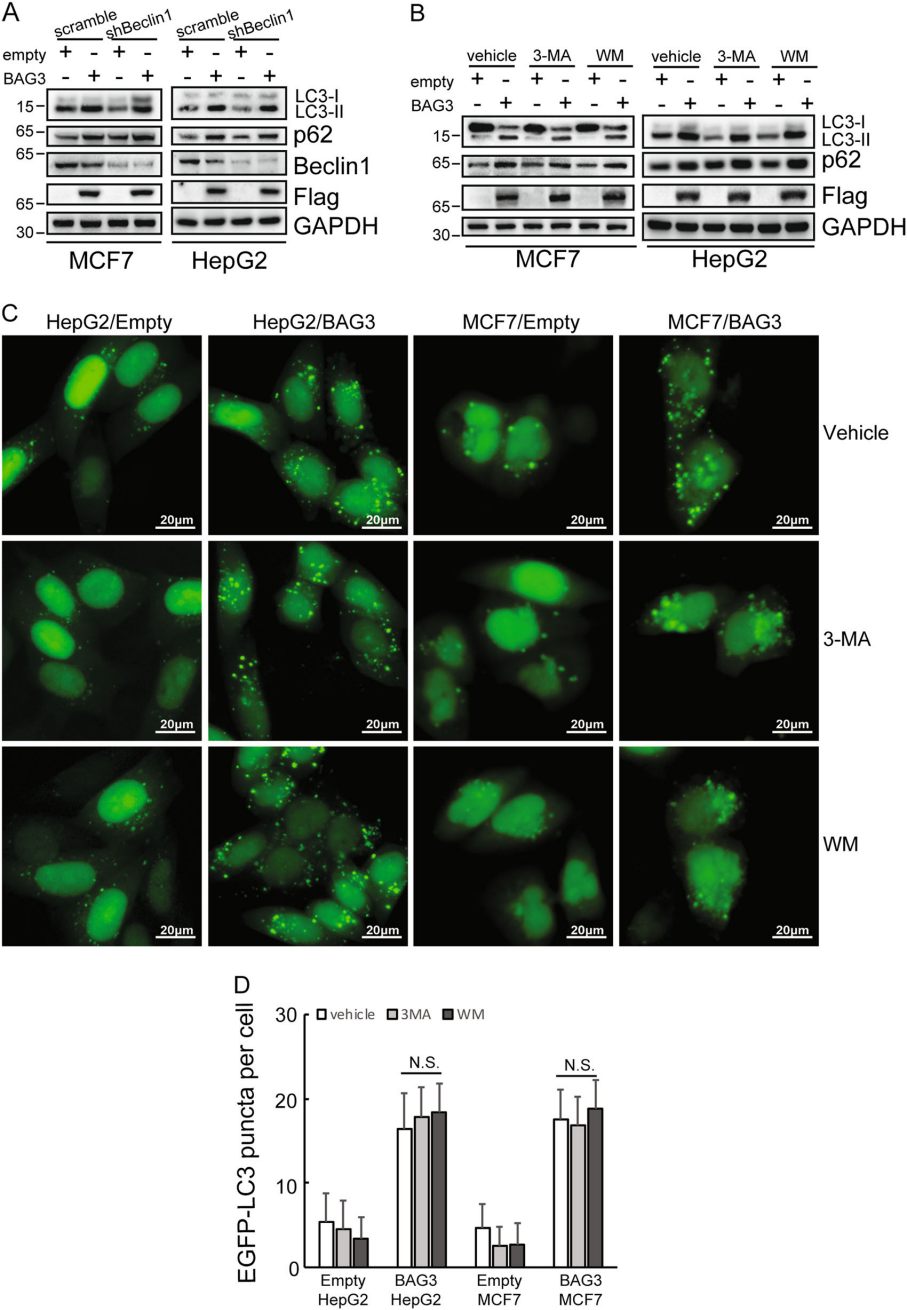
BAG3 overexpression induces autophagy independent of Beclin 1 and PtdIns3K. **a** Control or BAG3-overexpressing HepG2 and MCF7 cells were transfected with scramble shRNA or shRNA specific against Beclin1 (shBeclin1), and Western blot analysis was performed using the indicated antibodies. **b** HepG2 or MCF7 cells transduced with lentivirus containing empty or BAG3 construct were treated with vehicle, 3-MA or WM respectively, protein expression levels of LC3, p62 and BAG3 were analyzed using Western blot analysis. **c** HepG2 or MCF7 cells stably overexpressing EGFP-LC3B were infected with lentivirus containing empty or BAG3 construct. Then cells were treated with vehicle, 3-MA or WM, and the punctate distribution of EGFP-LC3B was visualized under the florescence microscopy. **d** Quantitative analysis of (**c**). Results shown represent mean ± SD from five representative microscopic fields and three independent experiments were performed. *P < 0.05. *N.S.* not significant. Error bars indicate means ± SD

BAG3 overexpression decreased Beclin 1 expression (Fig. 1a). In addition, we have reported implication of BAG3 in noncanonical autophagy induced by proteasome inhibition, which is independent of Beclin 1 and class III phosphatidylinositol 3-kinase (PtdIns3K) complex^8^. These data urged us to explore whether autophagy triggered by BAG3 is noncanonical. Knockdown of Beclin 1 unaltered autophagy activation mediated by BAG3 overexpression (Fig. 2a). In addition, neither 3-methyladenine (3-MA) nor wortmannin (WM), the pharmacological inhibitors of PtdIns3K, could suppressed transition of LC3-II (Fig. 2b), as well as puncta distribution of EGFP-LC3B (Fig. 2c, d) induced by BAG3 overexpression.

### BAG3 promotes autophagy via upregulation of glutaminase (GLS)

**Fig. 3:**
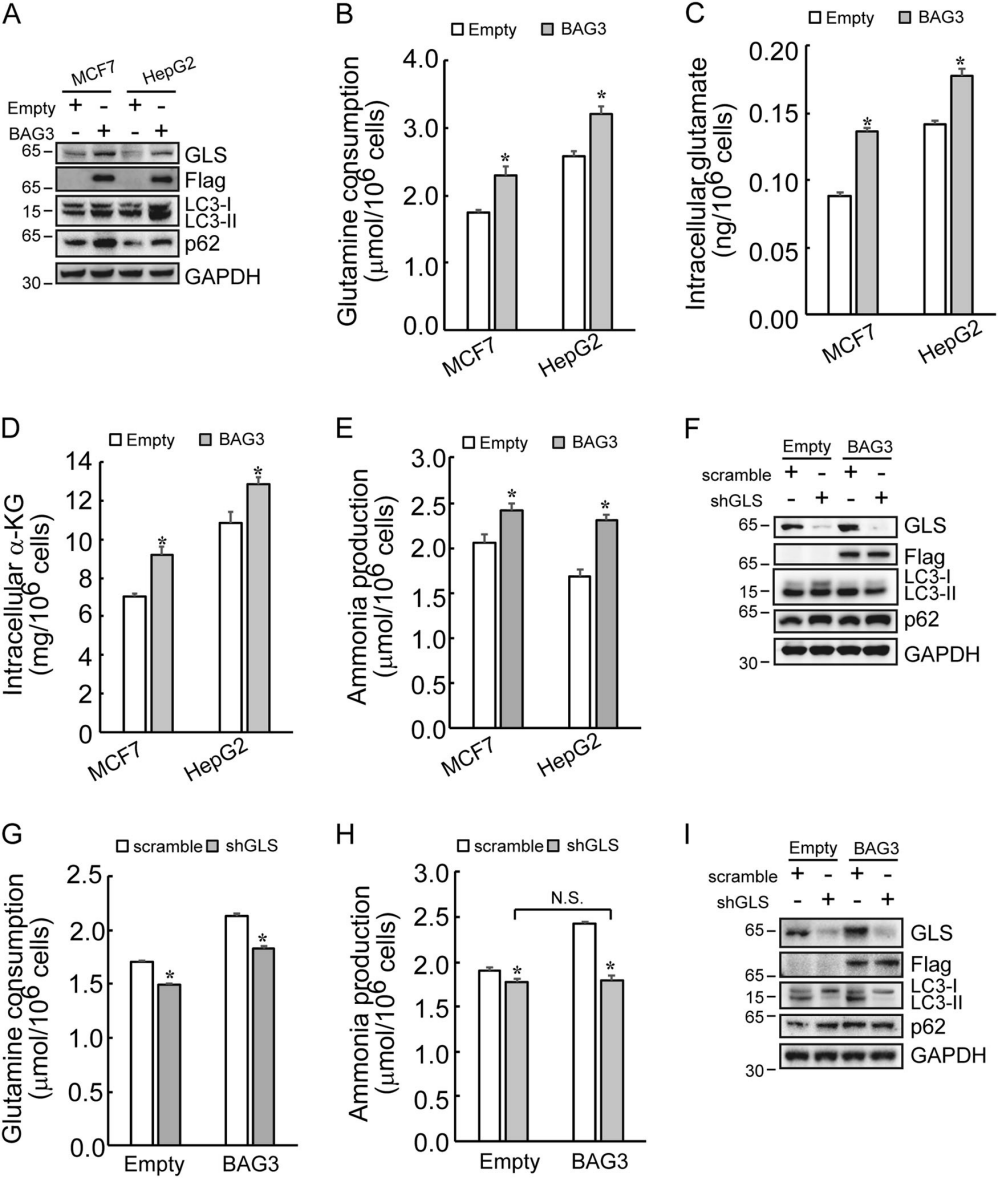
BAG3 promotes autophagy via upregulation of GLS. **a** Western blot analysis of changes induced by BAG3 overexpression in GLS, LC3 conversion and p62 levels in indicated cells. **b–e** HepG2 or MCF7 cells were infected with lentivirus containing empty or BAG3 construct, and glutamine consumption (**b**), intracellular glutamate production (**c**), intracellular α-KG (**d**), and ammonia production (**e**) were analyzed using the colorimetric method. **f** Control or BAG3-overexpressing HepG2 cells were transfected with scramble shRNA or shRNA specific against GLS (shGLS), and Western blot analysis was performed using the indicated antibodies. **g** and **h** Control or BAG3-overexpressing HepG2 cells were transfected with scramble shRNA or shRNA specific against GLS (shGLS), and glutamine consumption (**g**), ammonia production (**h**) were analyzed using the colorimetric method. **i** Control or BAG3-overexpressing MCF7 cells were transfected with scramble shRNA or shRNA specific against GLS (shGLS), and Western blot analysis was performed using the indicated antibodies. *P < 0.05. *N.S.* not significant. Error bars indicate means ± SD

Differential proteomics identified that BAG3 overexpression increased GLS (glutaminase) expression in MCF7 cells (Tables S1–S2; Supplemental Information). GLS is a key enzyme involved in glutaminolysis. In addition, ammonia derived from glutaminolysis induces autophagy^33,35^. For these considerations we reasoned that BAG3 might induce autophagy via upregulation of GLS. Western blot confirmed that BAG3 overexpression increased GLS expression in MCF7 and HepG2 cells (Fig. 3a). BAG3 cells consumed significantly more glutamine (Fig. 3b). Consistent with increased glutamine uptake, intracellular glutamate (Fig. 3c) and α-KG (Fig. 3d), a critical metabolite immediately downstream of glutamate, were also significantly increased in cells with ectopic BAG3 expression. Furthermore, BAG3 overexpression increased ammonia accumulation in culture media (Fig. 3e). To further confirm the role of GLS, GLS was knocked down using shRNA specific against GLS (shGLS). Knockdown of GLS significantly suppressed LC3-II accumulation mediated by BAG3 in HepG2 cells (Fig. 3f). In addition, GLS knockdown significantly blocked increase in glutamine consumption (Fig. 3g) and ammonia production (Fig. 3h) mediated by BAG3 overexpression in HepG2 cells. Importantly, glutamine consumption (Fig. 3g) and ammonia accumulation (Fig. 3h) were reduced to the same extent in control cells as well as in BAG3 overexpressing cells by GLS knockdown. Similar like in HepG2 cells, knockdown of GLS also significantly suppressed LC3-II accumulation induced by BAG3 in MCF7 cells (Fig. 3i).

### BAG3 stabilizes GLS by suppression its proteasomal degradation

**Fig. 4:**
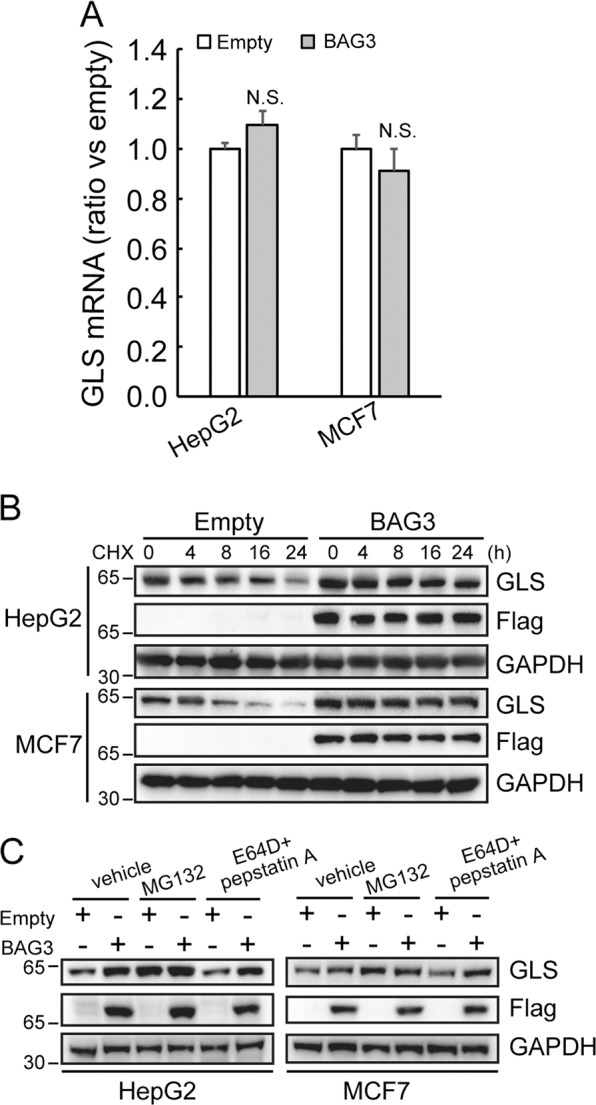
**BAG3 stabilizes GLS by suppression its proteasomal degradation.** **a** HepG2 or MCF7 cells were infected with lentivirus containing empty or BAG3 construct, and GLS mRNA expression was measured using RT-qPCR. **b** The effect of overexpressing BAG3 on the half-life of GLS was evaluated in indicated cells treated with cyclohexamide (CHX). **c** Control or BAG3-overexpressing HepG2 and MCF7 cells were treated with vehicle, MG132 or E-64d plus pepstatin A respectively, GLS protein expression was investigated using Western blotting. *P < 0.05. *N.S.* not significant. Error bars indicate means ± SD

Real-time PCR demonstrated that GLS mRNA expression level was unaltered by ectopic BAG3 expression (Fig. 4a), indicating that BAG3 increases GLS1 expression at the protein level. Blockade of novel protein synthesis by cycloheximide (CHX) demonstrated that ectopic BAG3 expression increased stability of GLS (Fig. 4b). MG132 and E64D/pepstatin A were then used to block proteasomal and lysosomal protein degradation, respectively. Compared with their control partner, cells with forced BAG3 expression expressed comparable level of GLS in the presence of MG132 (Fig. 4c).

### Ectopic BAG3 expression increases GLS succinylation at Lys158 and Lys164 sites

**Fig. 5:**
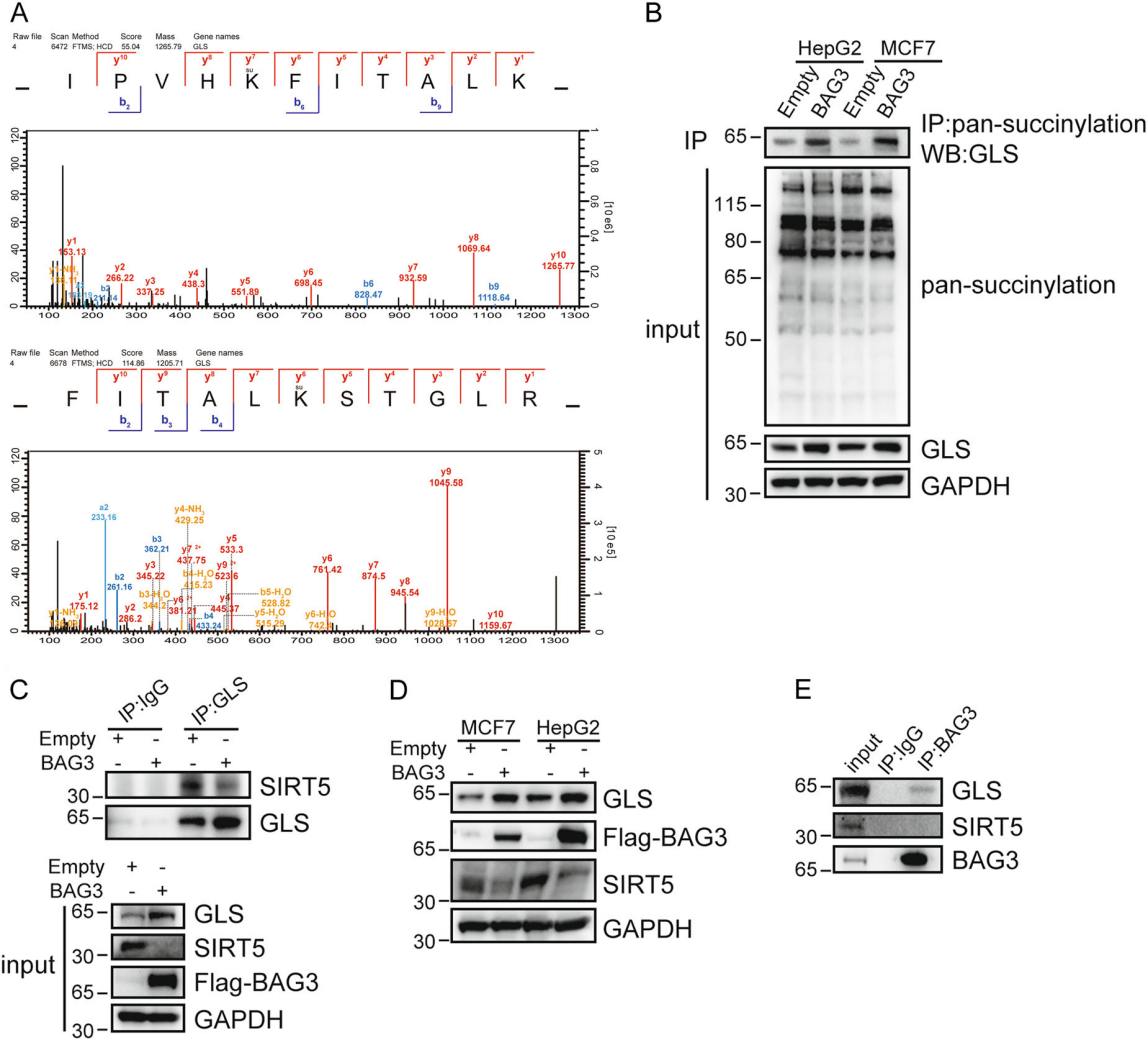
Ectopic BAG3 expression increases GLS succinylation at Lys158 and Lys164 sites. **a** Globally screening succinylation proteomics identified that lysine 158 and lysine 164 are succinylated in succinylated GLS. **b** BAG3 overexpression leads to increased succinylation of GLS in HepG2 and MCF7 cells. **c** Immunoprecipitation assays of interaction between GLS and SIRT5 in HepG2 cells infected with lentivirus containing empty or BAG3 construct. Ectopic BAG3 expression decreases the interaction between GLS and SIRT5. **d** Western blot analysis of the levels of SIRT5 in control or BAG3-overexpressing cells. **e** Co-immunoprecipitation assays of interaction between BAG3 and GLS or SIRT5 in HepG2 cells

Globally screening succinylation proteomics identified that GLS was succinylated at K158 and K164 sites, which was increased in MCF7 cells with ectopic BAG3 expression (Fig. 5a). It has been reported that GLS1 succinylation increases its stability and is responsible for ammonia-induced autophagy mediated by Sirtuin 5 (SIRT5) downregulation^35^. Total cellular succinylation levels were unaltered by BAG3 overexpression, while GLS succinylation levels were significantly increased in MCF7 and HepG2 cells (Fig. 5b). It has been reported that SIRT5 is responsible for desuccinylation of GLS^35^, immunoprecipitation (IP) was then performed. Interaction between GLS and SIRT5 was suppressed by BAG3 in HepG2 cells (Fig. 5c). Unexpectedly, we demonstrated that BAG3 overexpression decreased SIRT5 expression in HepG2 cells (Fig. 5d). SIRT5 expression was also decreased by ectopic BAG3 expression in MCF7 cells (Fig. 5d). Furthermore, Co-IP demonstrated that BAG3 interacted with GLS, but not SIRT5 (Fig. 5e).

### Succinylation at Lys158 and Lys164 sites stabilizes GLS via suppression its Lys48-linked ubiquitination

**Fig. 6:**
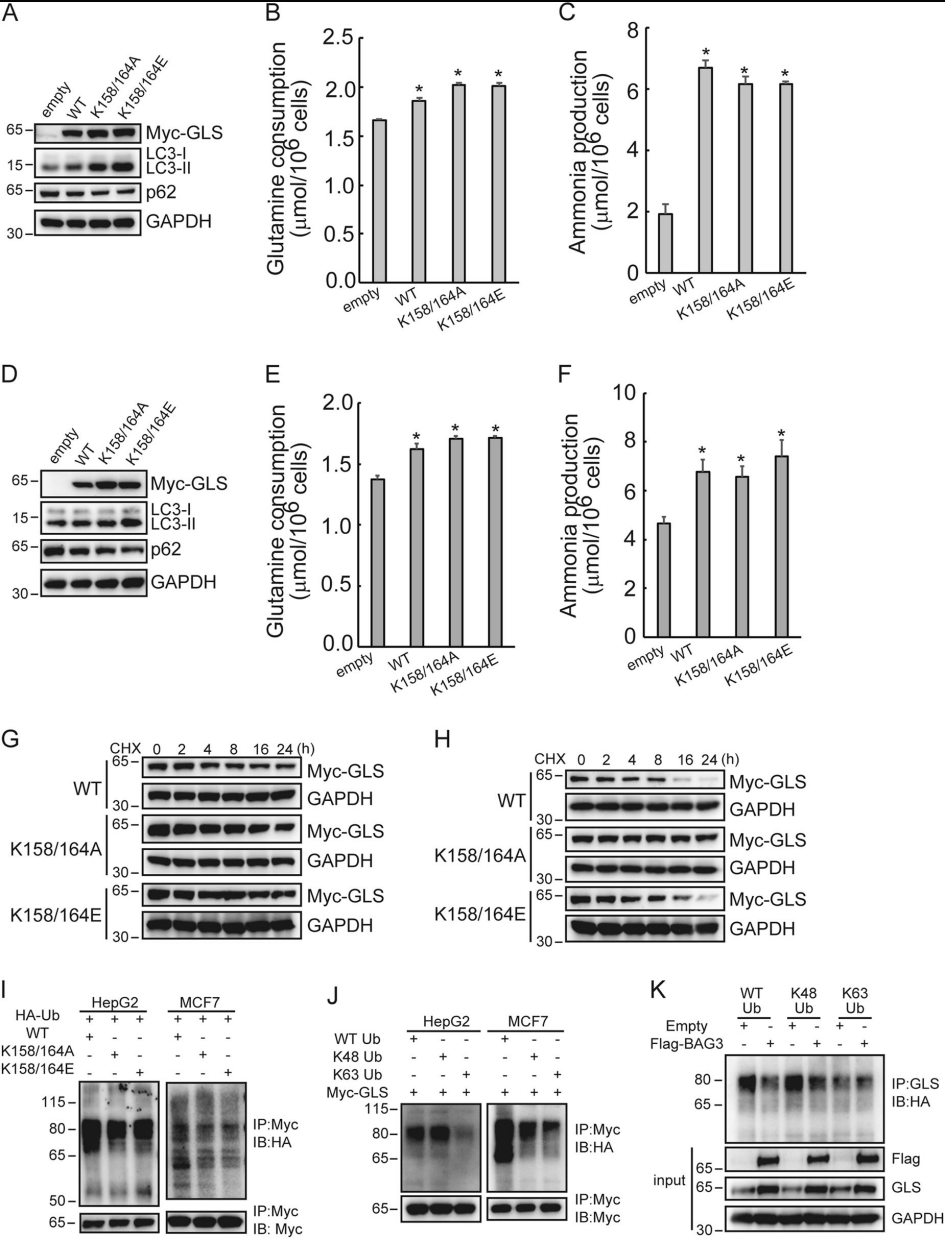
Succinylation at Lys158 and Lys164 sites stabilizes GLS via suppression its Lys48-linked ubiquitination. **a–c** HepG2 cells were infected with lentivirus containing empty, GLS (WT), GLS (K158/164 A) or GLS (K158/164E) construct. **a** Western blot analysis was performed using the indicated antibodies. Glutamine consumption (**b**) and ammonia production (**c**) were analyzed using the colorimetric method. **d**–**f** MCF7 cells were infected with lentivirus containing empty, GLS (WT), GLS (K158/164 A) or GLS (K158/164E) construct. **d** Western blot analysis was performed using the indicated antibodies. Glutamine consumption (**e**) and ammonia production (**f**) were analyzed using the colorimetric method. **g** and **h** The effect of overexpressing GLS (WT), GLS (K158/164 A) or GLS (K158/164E) on the half-life of GLS was investigated in indicated cells treated with cyclohexamide (CHX). **i** In vivo ubiquitination was performed by transfecting HepG2 or MCF7 cells with indicated expression plasmids. Cells were transfected with the indicated constructs for 36 h and incubated with MG132 for additional 4 h. Lysates were subjected to denaturing immunoprecipitation with an anti-Myc antibody and the levels of GLS (WT), GLS (K158/164 A) or GLS (K158/164E) ubiquitination were detected by immunoblotting with anti-HA antibody. **j** GLS polyubiquitination assay following transfection of HA-tagged ubiquitin (Ub) or its mutant forms K48 and K63. **k** Control or BAG3-overexpressing HepG2 cells were transfected with HA-Ub or its mutant forms K48 and K63, and analyzed by immunoprecipitation with an anti-Myc antibody and immunoblotting with the indicated antibodies. *P < 0.05. Error bars indicate means ± SD

Vectors containing wild type (WT), nonsuccinylation mutant K158/164 A (in which Lys158 and Lys164 was replaced by Ala), and succinylation mimic mutant K158/164E (in which Lys158 and Lys164 were replaced by Glu) GLS were then constructed. Unexpectedly, WT, nonsuccinylation mutant K158/K164A, and succinylation mimic mutant K158/K164E GLS significantly activated autophagy in HepG2 cells (Fig. 6a). In addition, WT, K158/164 A, and K158/K164E GLS significantly increased glutamine consumption (Fig. 6b) and ammonia production (Fig. 6c) in HepG2 cells. Similarly, WT, K158/164 A, and K158/K164E GLS increased autophagy (Fig. 6d), glutamine consumption (Fig. 6e), and ammonia production (Fig. 6f) in MCF7 cells. It should be noted that both succinylation mimic and nonsuccinylation mutant GLS demonstrated more higher expression level than wild type GLS (Fig. 6a, d). Blockade of novel protein synthesis by CHX demonstrated that both succinylation mimic and nonsuccinylation mutation increased stability of GLS protein in HepG2 (Fig. 6g) and MCF7 (Fig. 6h) cells. In vivo ubiquitination assays demonstrated that both succinylation mimic and nonsuccinylation mutation reduced ubiquitination levels of GLS in HepG2 and MCF7 cells (Fig. 6i). Using Lys48 (K48) or Lys63 (K63) alone capable ubiquitination mutant ubiquitin demonstrated that GLS was mainly ubiquitinated through Lys48 (Fig. 6j). Importantly, BAG3 significantly suppressed K48-linked ubiquitination of GLS (Fig. 6k).

## Discussion

BAG3 is a multifunctional protein and its involvement in regulation of autophagy receives specific attention recently. BAG3 is widely reported to be a stimulator of autophagy, and especially is critical for the selectivity of autophagy homeostasis^7,8,10,13,17–22^. However, the molecular mechanism(s) underlying regulation of autophagy by BAG3 are not yet fully elucidated. We have reported that BAG3 plays a critical role in Beclin 1- and PtdIns3K-independent noncanonical autophagy under proteasome inhibition previously^8^. The current study demonstrated that BAG3 also enhanced autophagic activity independent of Beclin 1 and PtdIns3K under nonstress condition. In the present study we demonstrated that BAG3 overexpression enhanced glutaminolysis via stabilizing of GLS. Both BAG3 and GLS are often highly expressed and play pro-survival activity in cancer cells. Our results showed that GLS expression and ammonia production were increased in BAG3-overexpression cells with a corresponding autophagy induction. Importantly, knockdown of GLS completely prevented ammonia accumulation and autophagy induction mediated by BAG3 overexpression, indicating that GLS stabilization might play a central role for increased ammonia production and autophagy induction in BAG3 overexpressing cells. Except for induction of autophagy, ammonia can also trigger apoptosis. In the current study, we demonstrated that ectopic BAG3 expression had no effects on viability of HepG2 or MCF7 cells (data not shown).

There are two GLS isoforms, the kidney-type GLS and the liver-type GLS2, which are encoded by the genes *GLS1* and *GLS2*, respectively. GLS catalyzes conversion of glutamine to glutamate and ammonia. GLS is often upregulated in tumors and is the main isoform responsible for glutaminolysis in tumor cells^27^. It has been reported that GLS expression or catalytic activity is regulated by a variety of oncogenes and survival signals in cancer cells at the multiple levels. Myc promotes glutaminolysis via induction of GLS expression at the transcriptional level in cancer cells^36,37^. GLS expression is also regulated at the protein stability level. For example, ubiquitin ligase APC/C (anaphase-promoting complex/cyclosome)-Cdh1 decreases GLS stability via promoting its ubiquitination and subsequent proteasomal degradation^38,39^. GLS is also succinylated at the post translational level, and succinylation increases its protein stability^35^. In addition, it is also reported that NF-κB and Raf-MEK-ERK signaling pathways regulate GLS catalytic activity without affecting its protein expression^40,41^. The current study demonstrated that BAG3 increased GLS protein stability by preventing its proteasomal degradation. We also identified that GLS was succinylated at Lys158 and Lys164 sites. The current study demonstrated that BAG3 might prohibit interaction of GLS with SIRT5 and decrease its desuccinylation via two mechanisms: BAG3 decreases SIRT5 expression, thereby globally decreasing desuccinylation of SIRT5 substrates including GLS, alternatively, BAG3 interacts with GLS, thereby prohibiting interaction of GLS with SIRT5. Our data showed that BAG3 decreased SIRT5 expression, but had no effect on total protein succinylation, indicating that other mechanism(s) rather than SIRT5 might be implicated in BAG3-mediated succinylation regulation. Thereby, our data indicated that BAG3 enforces GLS succinylation specifically via both SIRT5 reduction and interaction with GLS.

It has reported that GLS is subjected to succinylation, which increases its protein stability^35^. The current study demonstrated that both succinylation mimic and nonsuccinylation mutation increased GLS stability, indicating that not succinylation per se, but occupation at Lys158 and Lys164 sites might be critical for its stabilization. Indeed, we found that ubiquitination level of GLS was reduced by both succinylation mimic and nonsuccinylation mutation at Lys158 and Lys164 sites. Therefore, the current study indicates that occupation of Lys158 and Lys164 sites by succinyl group prohibits addition of ubiquitin molecule, thereby increases GLS stability via preventing its ubiquitination and subsequent proteasomal degradation.

In sum, to our knowledge, our study for the first time demonstrates that succinylation at Lys158 and Lys164 sites stabilizes GLS via preventing its ubiquitination and subsequent proteasomal degradation. In addition, our data show that BAG3 activates autophagy via stabilizing GLS and promoting glutaminolysis. Therefore, given the high relevance of glutaminolysis for proliferating cancer cells, as well as involvement of BAG3 in regulation of glutaminolysis, BAG3 may become a therapeutic optional target against cancer.

## Materials and methods

### Reagents and antibodies

The following biochemical reagents were used: E64D (Sigma-Aldrich, E8640), Pepstatin A (Sigma-Aldrich, P5318), MG132 (Sigma-Aldrich, M7449), 3-MA (Sigma-Aldrich, M9281), CQ (Sigma-Aldrich, C6628), Wortmannin (Sigma-Aldrich, 35441), Puromycin (Sigma-Aldrich, P2755), CHX (Abcam, ab120093).

Antibodies were used against the following: GLS (Sigma-Aldrich, WH0002744M1), GLS (Abcam, ab156876), GAPDH (Merck-Millipore, AB2302), LC3B (Origene, am20213pu), P62 (BD Biosciences, 610833), Beclin1 (Cell Signaling Technology, 3495), ATG3 (Cell Signaling Technology, 3415), ATG5 (Cell Signaling Technology, 12994), ATG7 (Cell Signaling Technology, 8558), ATG12 (Cell Signaling Technology, 4180), SIRT5 (Cell Signaling Technology, 8779), c-Myc (Invitrogen, R950-25), DYKDDDDK Tag (Cell Signaling Technology, 14793), HA-Tag (Cell Signaling Technology, 3724), pan-succinylation (PTM Biolabs, Hangzhou).

### Cell culture

HepG2 cell lines were maintained in Dulbecco’s Modified Eagle’s Medium (DMEM; Sigma-Aldrich, D6429), MCF7 cell lines were maintained in RPMI 1640 medium (Sigma-Aldrich, R8758). Both mediums were supplemented 10% fetal bovine serum (Sigma-Aldrich, F9665). Cells were maintained at 37 °C in a humidified atmosphere of 5% CO2 and 95% air.

### Lentiviral vector construction and recombinant lentivirus production

Gene encoding BAG3, GLS, GLS (K158/164 A) and GLS (K158/164E) was cloned into the lentiviral vector, pGC-LV-GV166 (GeneChem Co., Ltd., Shanghai, China). DNA sequencing was performed by GeneChem to verify the sequence of the insert, and the identities were 100%. Following construction, 293 T cells were contransfected with the recombined lentivirus vector, the pHelper 1.0 plasmid (Invitrogen, Waltham, MA) and the pHelper 2.0 plasmid using Lipofectamine 2000 (Invitrogen, Waltham, MA). Recombinant lentiviruses were harvested at 48 and 72 h post-transfection, centrifuged to get rid of cell debris, and then filtered through 0.22 μm cellulose acetate filter. Ultimately, a concentrated lentivirus solution was obtained, with a final titer of 1.0 × 10^9^ TU/L.

### Gene expression transferred by recombinant lentiviral infection

Cells were infected with recombinant virus expressing BAG3 tagged with Flag epitope (Flag-BAG3), and expressing GLS, GLS (K158/164 A) or GLS (K158/164E) tagged with Myc epitope (Myc-GLS) for 12 h. After 72 h, the infected cells were subjected to puromycin selection. Western blot was used to measure the infection efficiency of each gene and the negative control. Then cells were treated as indicated and subjected to further analysis.

### RNA isolation and Real-time qPCR

Total RNA was purified using Qiagen RNeasy Mini kit (Qiagen, 74106) and first strand cDNA was synthesized from 2 μg of total RNA using GoScript™ Reverse Transcription System (Promega, A5000) according to the manufacturer’s instructions. Real-time PCR analysis was performed in triplicate on the ABI prism 7000 sequence detection system (Applied Biosystems) using the GoTaq® qPCR and RT-qPCR Systems (Promega, A6001). For GLS, the forward primer was 5′- TGGGTATGATGTGCTGGTCTC-3′ and reverse was 5′- AAGGAATGCCTTTGATCACCAC-3′. For 18 S RNA, the forward primer was 5′-CGGACAGGATTGACAGATTGATAGC-3′ and reverse was 5′-TGCCAGAGTCTCGTTCGTTATCG-3′. Results were normalized against those of 18 S RNA and presented as ratio vs. vehicle-treated control.

### Measurement of glutamine consumption

Cells were plated in 24-well plate at the density of 5 × 10^4^ cells per well in 0.5 ml DMEM. A blank well without cells was included as the control. After 24 h incubation, glutamine concentrations in the culture medium were analyzed using Glutamine Detection Assay Kit (Abnova, KA1627). The levels of glutamine consumption were determined by subtracting the concentration of glutamine in the control medium from that in the sample medium. The numbers of cells in each well were counted and used to normalize the data.

### Glutamate assays

Intracellular glutamate was analyzed by an enzymatic assay and measured at 450 nm using Glutamate Assay Kit (Sigma, MAK004), following the manufacture’s instruction. The concentration of glutamate was normalized to total protein concentration which was measured by a BCA protein assay kit (Thermo Scientific).

### Ammonia assay

Ammonia concentration was measured by an ammonia assay kit (Sigma-Aldrich, AA0100) according to the manufacturer’s protocol. Briefly, Cell medium was mixed with ammonia assay reagent and incubated for 5 min at room temperature. Measure the absorbance of each sample at 340 nm. 10 μl of L-Glutamate Dehydrogenase solution was added and incubated for 5 min at room temperature. L-Glutamate dehydrogenase reacts specifically with ammonia. Then, the absorbance was read at 340 nm in a Biotek Cytation5 cell imaging multi-mode reader. The decrease in absorbance at 340 nm is proportional to the ammonia concentration.

### α-ketoglutarate assay

α-KG concentration was measured according to the manufacturer’s protocols (Sigma, MAK054). Briefly, 100 μl of ice-cold α-KG buffer was used to homogenized 1 × 10^6^ cells. Samples were centrifuged at 13,000×*g* for 10 min, adjusted to a final volume of 50 μl with α-KG assay buffer. To prevent interference from enzymes, samples should be deproteinized with a 10 kDa MWCO (Millipore) spin filter before addition to the reaction. 50 μL of the appropriate reaction mixture was added to each well and incubated at 37 °C for 30 min. All samples and standards were run in duplicate. The absorbance of each reaction system was measured at 570 nm in a Biotek Cytation5 cell imaging multi-mode reader.

### In vivo ubiquitination assay

MCF7 and HepG2 cells were transfected with HA-Ub and Myc-GLS or its mutant expression plasmids. The transfected cells were incubated for 36 h and then exposed to 10 μM MG132 treatment for additional 4 h. Cell lysates were subjected to denaturing immunoprecipitation with anti-Myc antibody, and the ubiquitination of GLS was detected with anti-HA antibody.

### Co-immunoprecipitation

Cell lysates were pre-cleared with Protein-A/G beads (biomake, B23201) and kept on a rotator for 1 h at 4 °C. Lysates were centrifuged and 2 μg of primary antibody or corresponding IgG was added to the precleared lysates and kept on a rotator overnight at 4 °C. After incubation, 25 μl of Protein-A/G beads was added and kept on a rotator for 2 h at 4 °C. The immunoprecipitates were washed three times with lysis buffer, then analyzed by Western blot analysis.

### Western blot analysis

Cells were washed with ice-cold PBS and lysed with a lysis buffer (20 mM Tris-HCl, 150 mM NaCl, 2 mM EDTA, 1% NP-40, and protease inhibitor cocktail) on ice. After 30 min, cell lysates were centrifuged at 14,000×*g* at 4 °C for 15 min, and protein concentration was quantified using the BCA protein assay kit (Thermo Scientific, 23225). Equivalent amounts of protein (25 μg) were separated using 10 or 12% SDS-PAGE and transferred to PVDF membranes (Merck-Millipore, IPVH00010). The membranes were blocked with 5% skim milk powder in Tris-buffered saline containing 0.1% Tween 20 at room temperature, and probed with primary antibody solution overnight at 4 °C. The membranes were washed three times with TBST and incubated for 1 h with an appropriate horseradish peroxidase (HRP)-conjugated secondary antibody (1:5000). Specific proteins were detected using ECL solutions (Merck-Millipore, WBKLS0500). All the experiments were performed three times, each time in triplicate.

### Transmission electron microscopy (TEM)

After fixation in 2.5% glutaraldehyde at 4 °C for at least 60 min, cells were postfixed in aqueous 1% OsO_4_ and 1% K_3_Fe(CN)_6_ for 1 h and dehydrated through a graded series of 30 to 100% ethanol. Cells were infiltrated and embedded in Polybed 812 epoxy resin (Polysciences, 02597–50). Ultrathin (60 nm) sections were collected on copper grids and stained with 2% uranyl acetate in 50% methanol for 10 min, followed by 1% lead citrate for 7 min. Sections were photographed using a JEOL JEM 1210 transmission electron microscope (JEOL) at 80 kV.

### Fluorescence microscopy

Cells were stably transfected with pcDNA3.1-EGFP-LC3B plasmid. Images of live cells were taken using Olympus IX71 fluorescence microscopy (Olympus) equipped with digital epifluorescence imaging.

### Small hairpin RNA (shRNA)

shRNAs against GLS and Beclin1 were purchased from Open Biosystems. Transfection of shRNA oligonucleotide was performed with Lipofectamine 2000 (Invitrogen, Waltham, MA) according to the manufacturer’s recommendations.

### Statistics

The statistical significance of the difference was analyzed by ANOVA and post hoc Dunnett’s test. Statistical significance was defined as *P* < 0.05. All experiments were repeated three times, and data were expressed as the mean ± SD (standard deviation) from a representative experiment.

## Supplementary information

Supplementary Table 1

Supplementary Table 2
