# Supplementary material for: BAG3 promotes autophagy and glutaminolysis via stabilizing glutaminase
Source: Cell Death Dis. 2019 Mar 25;10(4):284. doi: 10.1038/s41419-019-1504-6 (PMC6433946; doi:10.1038/s41419-019-1504-6)
Supplement: Supplementary file 1 — Supplementary Table 1 [file 41419_2019_1504_MOESM1_ESM.doc]

**Table 1:** Summary of identified and quantified sites and proteins

| **Name** | **Identified** | **Quantified** |
| --- | --- | --- |
| **Sites** | 1,460 | 1,368 |
| **Proteins** | 531 | 473 |

Note: *p*<0.05.
