# Supplementary material for: BAG3 promotes autophagy and glutaminolysis via stabilizing glutaminase
Source: Cell Death Dis. 2019 Mar 25;10(4):284. doi: 10.1038/s41419-019-1504-6 (PMC6433946; doi:10.1038/s41419-019-1504-6)
Supplement: Supplementary file 2 — Supplementary Table 2 [file 41419_2019_1504_MOESM2_ESM.doc]

**Table 2:** Summary of differentially quantified sites and proteins

| **Name** | **Up-regulated**  **(>1.5)** | **Down-regulated**  **(<0.67)** |
| --- | --- | --- |
| **Sites** | 99 | 115 |
| **Proteins** | 76 | 80 |

Note: *p*<0.05.
